# Molecular Beam Epitaxy of β-(In_*x*_Ga_1–*x*_)_2_O_3_ on β-Ga_2_O_3_ (010): Compositional Control, Layer Quality, Anisotropic Strain Relaxation, and Prospects for Two-Dimensional Electron Gas Confinement

**DOI:** 10.1021/acsami.3c19095

**Published:** 2024-02-29

**Authors:** Piero Mazzolini, Charlotte Wouters, Martin Albrecht, Andreas Falkenstein, Manfred Martin, Patrick Vogt, Oliver Bierwagen

**Affiliations:** † Paul-Drude-Institut für Festkörperelektronik, Leibniz-Institut im Forschungsverbund Berlin e.V., Hausvogteiplatz 5-7, 10117 Berlin, Germany; ‡ Leibniz-Institut für Kristallzüchtung, Max-Born-Str. 2, 12489 Berlin, Germany; § Institute of Physical Chemistry, RWTH Aachen University, D-52056 Aachen, Germany; ∥ Materials Department, University of California Santa Barbara, Santa Barbara, California 93106, United States

**Keywords:** β-Ga_2_O_3_ alloys, oxides semiconductor epitaxy, structural defects, compositional control, multilayer structure

## Abstract

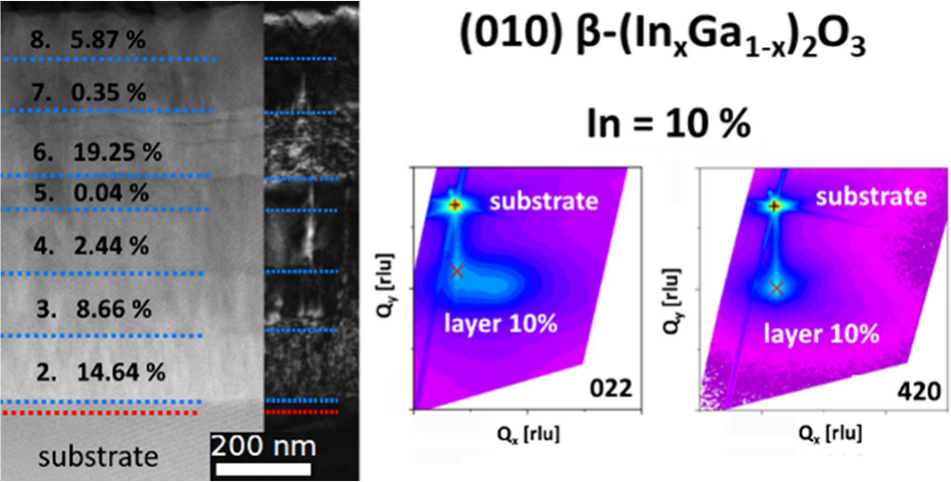

In this work, we investigate the growth of monoclinic β-(In_*x*_Ga_1–*x*_)_2_O_3_ alloys on top of (010) β-Ga_2_O_3_ substrates via plasma-assisted molecular beam epitaxy. In particular, using different *in situ* (reflection high-energy electron diffraction) and *ex situ* (atomic force microscopy, X-ray diffraction, time-of-flight secondary ion mass spectrometry, and transmission electron microscopy) characterization techniques, we discuss (i) the growth parameters that allow for In incorporation and (ii) the obtainable structural quality of the deposited layers as a function of the alloy composition. In particular, we give experimental evidence of the possibility of coherently growing (010) β-(In_*x*_Ga_1–*x*_)_2_O_3_ layers on β-Ga_2_O_3_ with good structural quality for *x* up to ≈ 0.1. Moreover, we show that the monoclinic structure of the underlying (010) β-Ga_2_O_3_ substrate can be preserved in the β-(In_*x*_Ga_1–*x*_)_2_O_3_ layers for wider concentrations of In (*x* ≤ 0.19). Nonetheless, the formation of a large amount of structural defects, like unexpected () oriented twin domains and partial segregation of In is suggested for *x* > 0.1. Strain relaxes anisotropically, maintaining an elastically strained unit cell along the a* direction vs plastic relaxation along the c* direction. This study provides important guidelines for the low-end side tunability of the energy bandgap of β-Ga_2_O_3_-based alloys and provides an estimate of its potential in increasing the confined carrier concentration of two-dimensional electron gases in β-(In_*x*_Ga_1–*x*_)_2_O_3_/(Al_*y*_Ga_1–*y*_)_2_O_3_ heterostructures.

## 1 Introduction

Gallium oxide has recently been earning large attention as a promising semiconductor for new-generation electronics. It has five different polymorphs,^1^ and because of its intrinsic ultrawide bandgap, its future application is especially related, but not limited, to the fields of power electronics^2,3^ and solar-blind UV photodetectors.^4^ In particular, its thermodynamically stable crystal phase—monoclinic β-Ga_2_O_3_—exhibits a bandgap of ≈ 4.7 eV,^5^ while its electronic conductivity can be controlled over a wide range throughout n-type extrinsic doping.^2^ Differently from the other polymorphs, bulk β-Ga_2_O_3_ can be grown from the melt.^6−8^ This allows to (i) have bulk material from semi-insulating to conducting with low structural defectivity and (ii) employ it after substrate fabrication for the thin film deposition of homo- or hetero- Ga_2_O_3_-based multilayer structures.

Similar to the AlGaN-InGaN system, Ga_2_O_3_ can be alloyed with Al_2_O_3_ and In_2_O_3_ virtually allowing for bandgap engineering of the material between ≈ 3 eV (low-end side of bixbyte In_2_O_3_) and ≈ 9 eV (high-end side of corundum α-Al_2_O_3_).^9^ The bandgap control in Ga_2_O_3_-based alloys enables charge carrier confinement at heterostructure interfaces, i.e., allowing the confinement of two-dimensional electron gases (2DEGs) that could help in the realization of high electron mobility transistors.^10^ In particular, a higher conduction band offset allows for the confinement of a higher sheet density of electrons in these 2DEGs. Nonetheless, the (In-Al-Ga)_2_O_3_ system has to account for different thermodynamically stable crystal structures of the binary end-members, setting up solubility limitations for the obtainable heterostructures. Due to the presence of bulk β-Ga_2_O_3_ substrates, the monoclinic crystal structure has been so far the only one capable of providing heterostructures with both low structural defectivity and surface roughness suitable for the obtainment of 2DEGs.^10−13^

In particular, 2DEG confinement has been experimentally demonstrated just at the β-(Al_*y*_Ga_1–*y*_)_2_O_3_/Ga_2_O_3_ interface of heterostructures deposited on (010)-oriented β-Ga_2_O_3_ substrates by both molecular beam epitaxy (MBE) and metal–organic vapor phase epitaxy (MOVPE) techniques.^10−13^ The β-Ga_2_O_3_ substrate orientation and offcut are fundamental factors for the deposition of Ga_2_O_3_ homo-^14−20^ or heterostructures^10−13,21,22^ with (i) low structural defectivity, (ii) smooth surfaces, and (iii) comparably high growth rates (in the case of MBE). So far, the (010) orientation has been the only one capable of meeting all these requirements, although also other orientations like offcut (100)^17,18,20,23^ and (001)^17,24^ have recently demonstrated similar potential. Particular effort has been put towards the realization of (010) heterostructures with larger band offsets by increasing the Al incorporation into β-(Al_*y*_Ga_1–*y*_)_2_O_3_ layers. So far, an Al concentration as high as *y* = 0.26 was reported for δ-doped β-(Al_*y*_Ga_1–*y*_)_2_O_3_/Ga_2_O_3_ heterostructures deposited via MOVPE on (010)-oriented substrates. This structure confined a total sheet electron concentration [2DEG + potential contribution from the δ-doped β-(Al_*y*_Ga_1–*y*_)_2_O_3_] of 6.4 × 10^12^ cm^–2^.^13^

On the other hand, the lower bandgap side of the β-Ga_2_O_3_ based alloys, i.e., β-(In_*x*_Ga_1–*x*_)_2_O_3_, has not been thoroughly examined so far. In particular, just few works reported on (2̅01)-oriented β-(In_*x*_Ga_1–*x*_)_2_O_3_ layers grown on c-plane sapphire [Al_2_O_3_(0001)] substrates.^25−28^ The layer quality on this substrate/orientation is most likely not compatible with the obtainment of 2DEGs due to the presence of a large amount of structural defects and high surface roughness^17^ at least without considering the employment of proper substrate offcuts to suppress the formation of rotational domains.^29^ To the best of our knowledge, very few experimental works^22,30^ reported on the structural quality and alloy composition obtainable for β-(In_*x*_Ga_1–*x*_)_2_O_3_ layers deposited on β-Ga_2_O_3_ substrates, and their investigation was limited to the (100) growth orientation. Based on the theoretical prediction that the conduction band minimum of β-(In_*x*_Ga_1–*x*_)_2_O_3_ is lower than that of β-Ga_2_O_3_,^31−33^ we believe that the realization of good quality β-(In_*x*_Ga_1–*x*_)_2_O_3_ layers could help to realize larger conduction band offsets in β-(In_*x*_Ga_1–*x*_)_2_O_3_/(Al_*y*_Ga_1–*y*_)_2_O_3_ heterostructures compared to the β-Ga_2_O_3_/(Al_*y*_Ga_1–*y*_)_2_O_3_ ones for the confinement of 2DEGs with higher sheet electron concentrations. Toward this goal, the present work aims at providing experimental evidence on (i) the deposition parameters that allow for In incorporation and (ii) the structural quality and phase purity as a function of *x* in β-(In_*x*_Ga_1–*x*_)_2_O_3_ thin films deposited on (010) β-Ga_2_O_3_ substrates via plasma-assisted MBE (PAMBE).

Maccioni et al.^31,32^ as well as Peelaers et al.^33^ theoretically predict In to replace Ga in the octahedral sites of the monoclinic structure, therefore suggesting a solubility limit of *x* = 0.5 for the β-(In_*x*_Ga_1–*x*_)_2_O_3_ alloy. An energetically favorable phase mix (monoclinic + bixbyite) has been, however, predicted for In concentrations exceeding 10–20 cation %.^31,32^ Experimentally, single phase β-(In_*x*_Ga_1–*x*_)_2_O_3_ powders were synthesized via solid-state reactions for *x* up to 0.44.^34^ Phase-pure monoclinic ()-oriented thin films with *x* up to ≈ 0.1 were MOVPE-grown on top of Al_2_O_3_(0001) substrates,^25^ while the pulsed laser deposition technique allowed to widen *x* up to ≈ 0.2 for layers deposited on the same substrate.^26,35^ Similarly, Oshima et al.^27^ investigated the growth of ()-oriented β-(In_*x*_Ga_1–*x*_)_2_O_3_ thin films on Al_2_O_3_(0001) with PAMBE reporting monoclinic phase-pure layers for *x* up to ≈ 0.3; nonetheless, the crystallinity of the layers (evaluated from X-ray diffraction, XRD) was reported to worsen while increasing the amount of incorporated In. Just from the XRD 2θ-ω scan reported in ref (27), however, it is not possible to clarify if the crystal phase obtained from the MBE growth of the (In_*x*_Ga_1–*x*_)_2_O_3_ is indeed monoclinic or if the presence of In triggered the formation of the orthorhombic κ polymorph whose {001} diffraction peaks are found to be at similar positions with respect to the () monoclinic structure.^36,37^ This is because the presence of an additional In^38^ or Sn^39^ flux during the MBE growth of Ga_2_O_3_ layers has been found to (i) widen the growth window of Ga_2_O_3_ toward higher substrate temperatures and (ii) stabilize the κ polymorph for heteroepitaxial films on Al_2_O_3_(0001).

The underlying mechanism has been described as metal exchange catalysis (MEXCAT) or metal-oxide-catalyzed epitaxy (MOCATAXY) in which the catalyst element (Sn/In) or its suboxide (SnO, In_2_O) is preferably oxidized on the growth surface and exchanged subsequently by Ga due to the stronger Ga–O than catalyst-O bonds, thus forming the Ga_2_O_3_ layer.^24,38−41^ This metal exchange leads to a segregation and/or desorption of the catalyst during the growth process, with the possibility to deposit α-,^42^ β-,^14,17,20,24^ and κ-Ga_2_O_3_ layers^38,39,43^ (depending on the used substrate) with just limited amounts of the catalyst being incorporated. Consequently, the incorporation of In for the formation of phase-pure β-(In_*x*_Ga_1–*x*_)_2_O_3_ alloys is particularly challenging in MBE. Vogt and Bierwagen^28^ gave experimental guidelines for the MBE deposition of (In_*x*_Ga_1–*x*_)_2_O_3_ alloys on Al_2_O_3_(0001) substrates, pointing out that lower substrate temperatures and/or higher oxygen fluxes allow for the kinetically driven incorporation of larger amounts of In; nonetheless, in that work no details about the obtained crystal phase (i.e., β and/or κ) and quality of the deposited alloyed layers were given. Recently, Ardenghi et al.,^44^ have experimentally shown that the stabilization of the β phase upon MEXCAT is mostly related to the overcome of the *T*-stability window of the metastable κ polymorph, i.e., for growth temperatures *T*_g_ ≥ 700 °C just monoclinic layers are synthesized despite the employment of the catalyst element and use of Al_2_O_3_(0001) substrate.

The present work starts from these experimental findings and aims at clarifying the possibility of depositing high crystal quality β-(In_*x*_Ga_1–*x*_)_2_O_3_ layers on β-Ga_2_O_3_ substrates by MEXCAT MBE/MOCATAXY with a focus on the (010) surface orientation. The reported results are discussed in view of its possible application in β-(In_*x*_Ga_1–*x*_)_2_O_3_/(Al_*y*_Ga_1–*y*_)_2_O_3_ heterostructures for the improved confinement of 2DEGs.

## 2 Experimental Section

**Figure 1:**
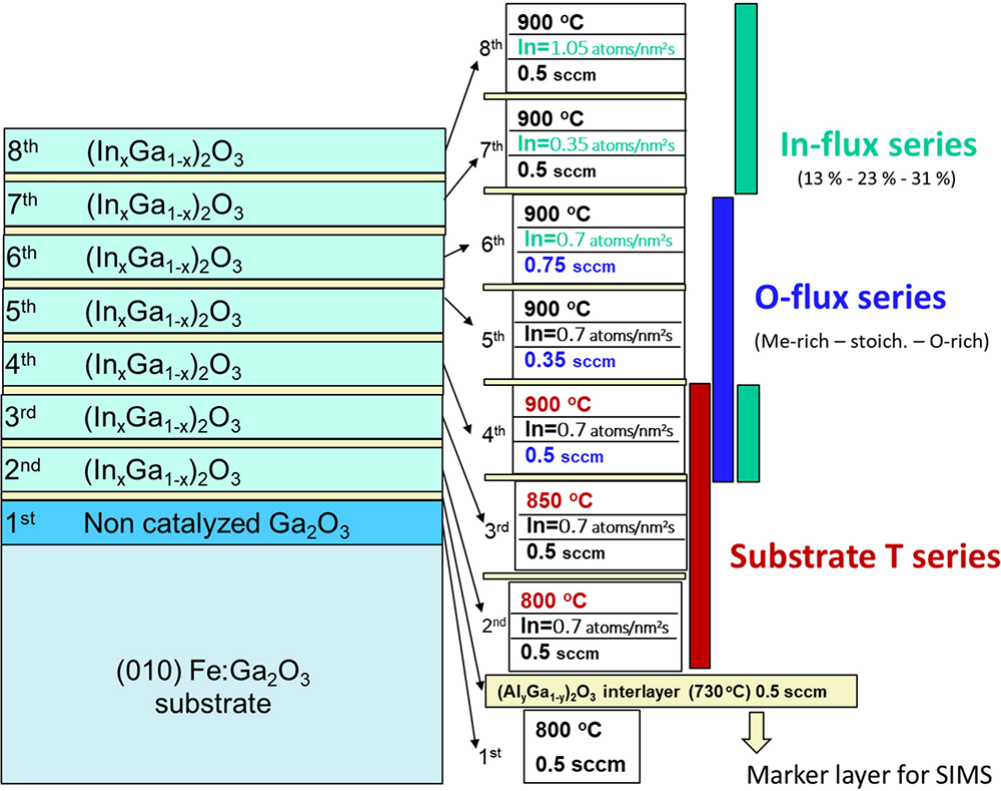
Exemplary drawing of the experimental design for the multilayer structure and of the investigated growth parameters (fixed Ga-flux of 2.34 Ga nm^–2^ s^–1^, plasma power of 300 W and varied substrate temperature, O-flux, and In-flux as indicated).

In order to provide rapid, efficient, and economic screening of growth parameters, a multilayer sample (sample PM) consisting of seven different β-(In_*x*_Ga_1–*x*_)_2_O_3_ layers has been deposited on top of an Fe-doped (010)-oriented β-Ga_2_O_3_ substrate via PAMBE. Its growth sequence is schematically shown in Figure 1 and details on the experimental setup and substrate preparation are given in ref (14). Ga and In-fluxes were measured by an ion-gauge as beam equivalent pressure (BEP) and converted into particle flux (nm^–2^ s^–1^) by measuring the respective oxide growth rate under conditions of full cation incorporation as described in refs.^14,21,40^ The Ga-flux, plasma power, and deposition time for the β-(In_*x*_Ga_1–*x*_)_2_O_3_ layers in PM were fixed at Φ_Ga_ = 2.34 nm^–2^ s^–1^, 300 W, and *t* = 30 min, respectively. For the investigation of the effect of deposition parameters, we varied the O-flow (0.35, 0.5, 0.75 standard cubic centimeter per minute [sccm]), substrate temperature (*T*_g_ = 800, 850, 900 °C), and In-flux [Φ_In_ = 0.35, 0.7, and 1.05 atoms/(nm^2^ s), corresponding to a nominal In concentration *x*_nom_ = Φ_In_ /(Φ_Ga_ + Φ_Ga_) = 0.13, 0.23, 0.31] indicated by red, blue, and light green bars, respectively, in Figure 1. β-(Al_*y*_Ga_1–*y*_)_2_O_3_ tracing/marker layers, indicated by yellow horizontal bars in Figure 1, were deposited between the β-(In_*x*_Ga_1–*x*_)_2_O_3_ to facilitate secondary ion mass spectrometry (SIMS) investigation. These layers were deposited for *t* = 80 s at *T*_g_ = 730 °C, Al cell *T* = 1008 °C, and O*-*flow = 0.5 sccm, resulting in a nominal thickness of 2–5 nm and expected Al content of *y* ≈ 0.05 that is not expected to affect the structural quality of the stack.^17^

The multilayer sample was monitored *in situ* by reflection high-energy electron diffraction (RHEED) to assess the layer quality.

In addition, single (In_*x*_Ga_1–x_)_2_O_3_ layer samples (P02, P10, U09, U13, and U19) with In content between 2 and 19 cation % were deposited as summarized in Table 1. These single layers were deposited in two different PAMBE chambers indicated by the sample names starting with P (Paul Drude Institute) and U (University of California at Santa Barbara), with PDI also being the one in which the multilayer structure was deposited (PM). Both chambers are equipped with an apparatus similar to the one previously described. The oxygen plasma units of the two MBE apparatuses were SPECS PCS and VEECO Unibulb whose O-flow rates were controlled by mass-flow controllers and foreline pressure controllers for P and U, respectively. Further details for the growth chamber of samples U09–U19 can be found in ref (21).

**Table 1:** Overview of the Investigated β-(In_*x*_Ga_1–*x*_)_2_O_3_ Layers^a^

sample	GR [nm/min]	In content (SIMS) [%]	thickness [nm]	*b* [*A*°]	*T*_g_ [°C]	Ga BEP/flux Φ_Ga_ [10^–7^ mbar/nm^–2^ s^–1^]	In BEP/flux Φ_In_ [10^–7^ mbar/nm^–2^ s^–1^]	*x*_nom_ = Φ_In_ /(Φ_Ga_ + Φ_Ga_)	oxygen flow/foreline pressure/plasma power [sccm/Torr/W]
β-Ga_2_O_3_ substrate		0		3.040					
PM		0—19, see Figure 2	...		see Figure 1	1.9/2.34	see Figure 1		see Figure 1
PM_2	4.3 ± 0.2	14.64 ± 0.3	130 ± 6		800	1.9/2.34	1.3/0.7	0.23	0.5/–/300
PM_3	3.7 ± 0.2	8.66 ± 0.2	112 ± 5		850	1.9/2.34	1.3/0.7	0.23	0.5/–/300
PM_4	3.6 ± 0.2	2.44 ± 0.04	108 ± 5		900	1.9/2.34	1.3/0.7	0.23	0.5/–/300
PM_5	1.9 ± 0.1	0.04 ± 0.004	58 ± 4		900	1.9/2.34	1.3/0.7	0.23	0.35/–/300
PM_6	4.1 ± 0.2	19.25 ± 0.2	124 ± 5	...	900	1.9/2.34	1.3/0.7	0.23	0.75/–/300
PM_7	2.9 ± 0.2	0.35 ± 0.04	88 ± 5		900	1.9/2.34	0.65/0.35	0.13	0.5/–/300
PM_8	3.5 ± 0.1	5.87 ± 0.2	106 ± 4		900	1.9/2.34	1.95/1.05	0.31	0.5/–/300
P02	3.5 ± 0.1	2.48 ± 0.3	105 ± 2	3.047	800	1.9/2.34	1.3/0.7	0.23	0.33/–/300
P10	4.1 ± 0.1	10.05 ± 0.3	122 ± 2	3.082	850	1.9/2.34	1.3/0.7	0.23	0.5/–/300
U09	3.9 ± 0.1	9.12 ± 0.3	164 ± 3	3.069	950	1.1/2	2.3/2.5	0.56	–/60/200
U13	3.9 ± 0.1	13.04 ± 0.3	177 ± 4	3.084	900	1.1/2	1/1.1	0.35	–/60/200
U19	4.4 ± 0.1	19.48 ± 1.3	155 ± 3	3.10	900	1.1/2	1/1.1	0.35	–/80/200

a Determined growth rate (GR), In content, layer thickness, and out-of-plane lattice parameter *b* determined by HRXRD of the 020 reflex, as well as growth parameters (Ga- and In-beam equivalent pressures [BEP] and corresponding fluxes, oxygen flow rate or foreline pressure, and plasma power) are given. For the single layers, the number following the initial letter reflects the In amount rounded to integer cation % in the β-(In_*x*_Ga_1–*x*_)_2_O_3_ corresponding alloy. The In content determined by SIMS is the mean value in the plateaus regions of the analyzed layers; the corresponding uncertainties are related to the standard deviations. For the thickness of the layers (SIMS), the error has been set to 2% (crater depth from interference microscopy Veeco Instruments, Inc.); in the case of the PM multilayer, the error over the determined thickness has been also compensated according to the uncertainty level over the thickness of the β-(Al_*y*_Ga_1–*y*_)_2_O_3_ tracing/marker layers.

The surface morphology of all samples was scanned with atomic force microscopy (AFM; Bruker Dimension Edge) in the PeakForce tapping mode. Their In-content was determined with time-of-flight ToF-SIMS using ToF-SIMS IV from IONTOF GmbH. The quantitative ToF-SIMS calibration of the In concentration was obtained from pulsed laser deposited (In_*x*_Ga_1–x_)_2_O_3_ reference films on Al_2_O_3_(0001) substrates analyzed by energy-dispersive X-ray analysis as explained in detail in ref (17). XRD measurements (PANalytical X’Pert Pro MRD using Cu Kα radiation) were performed on all samples. Symmetric, out-of-plane 2θ-ω scans and ω-rocking curves of the 020 reflex along the c* (001) and a* (100) in-plane directions as well as reciprocal space maps of the 420 and 022 reflexes were performed to investigate the out-of-plane lattice parameter as well as the strain relaxation of the layers, respectively. The out-of-plane lattice parameters were additionally measured by high-resolution XRD (HRXRD). Cross-sectional transmission electron microscopy (TEM—aberration-corrected FEI Titan 80–300 operating at 300 kV) along the [001] direction was carried out on the multilayer sample. Scanning TEM (STEM) images were recorded with a high-angle annular dark-field (HAADF) detector with an inner acceptance angle of 35 mrad and a camera length of 196 mm.

## 3 Results and Discussion

### 3.1 Indium Incorporation

**Figure 2:**
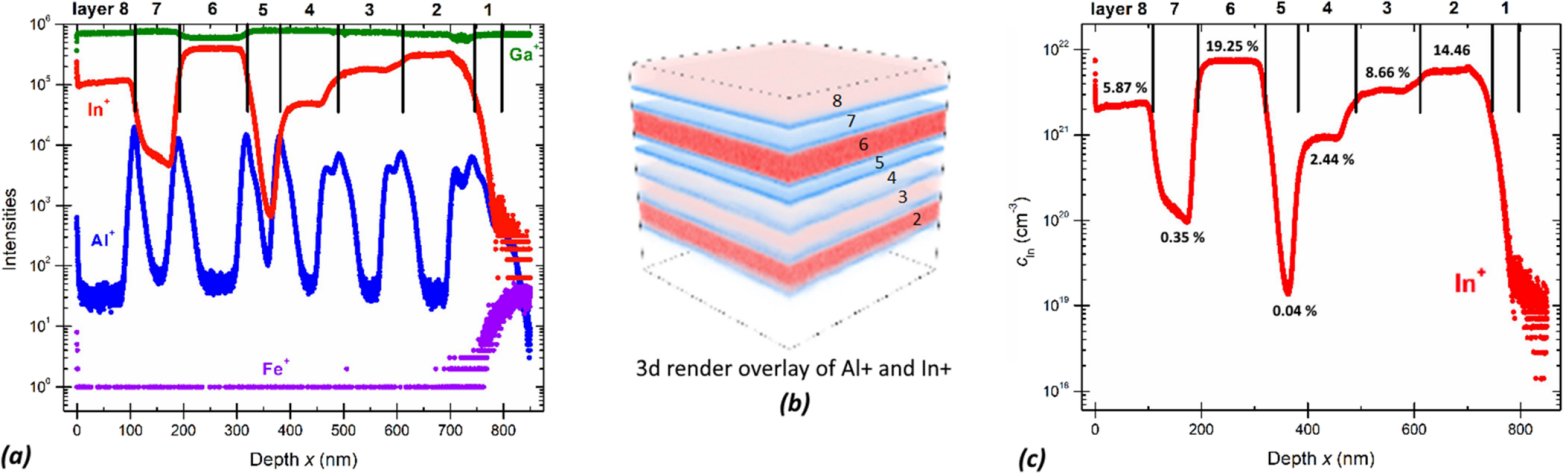
ToF-SIMS of the multilayer sample PM: (a) depth profile of Ga, In, Al, and Fe secondary ions, (b) 3d render overlay of a 100 × 100 μm area indicating In (red) and Al (blue) secondary ions, and (c) calibrated In concentration profile.

The multielemental intensity signals collected via ToF-SIMS in depth profiling for the multilayer (Figure 2a) allow us to distinguish all the eight deposited layers (sample PM). Apart from the different In^+^ signal collected for the individual layers, the presence of the β-(Al_*y*_Ga_1–*y*_)_2_O_3_ markers helps to better define the respective thicknesses (Figure 2a,b). The presence of a wider Al^+^ intensity distribution for deeper probing depths should be ascribed to the roughening of the layer throughout the sputtering process (Figure 2a).

**Figure 3:**
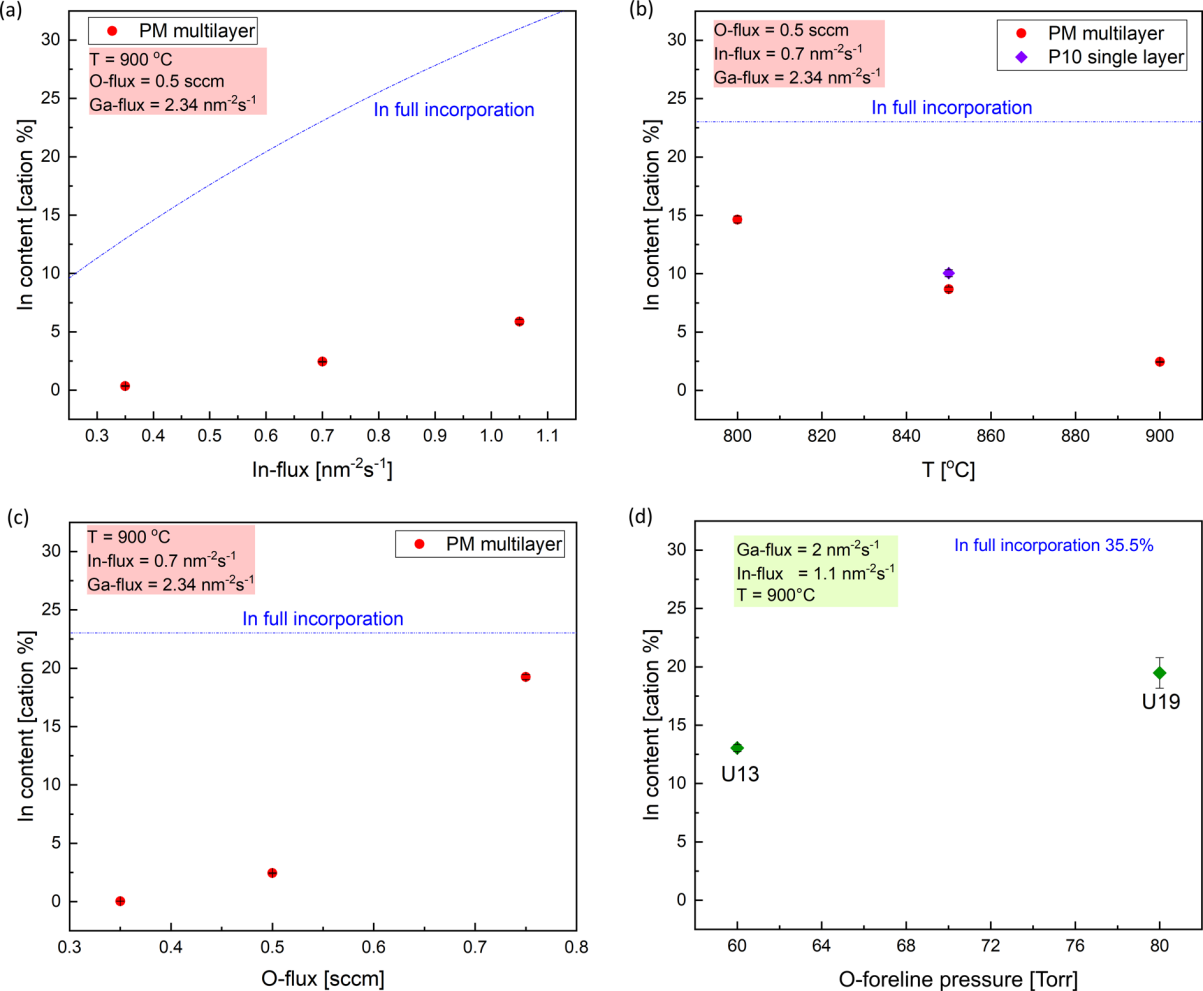
Dependence of In content on to the provided (a) In-flux, (b) substrate *T*, and (c) O_2_-flux (plasma *P* = 300 W)/(d) O_2_-foreline pressure (plasma *P* = 200 W) for the β-(In_*x*_Ga_1–*x*_)_2_O_3_ multilayer (PM disks) and single layers (squares, violet PDI and green UCSB). The full set of data is reported in Table 1.

The In concentrations (evaluated as cationic %) obtained in the multilayer structure span from a minimum of 0.04 to a maximum of 19.25%. Using these SIMS results, under the hypothesis that all of the detected In is present as Ga substitutional (i.e., In_Ga_), we report in Figure 3a–c (red circles) the In incorporation dependence with the main three tested synthesis parameters (see Figure 1) during the multilayer deposition. In Figure 3a, we identify a monotonic increase in In with increasing In-flux (fixed *T*_g_ = 900 °C and 0.5 sccm). Nonetheless, under such fixed growth conditions, the maximum In incorporation was limited to 5.87% (i.e., low incorporation efficiency). The substrate temperature plays an important role in the In-flux incorporation (fixed O-flux = 0.5 sccm and In-flux = 0.7 nm^–2^ s^–1^, Figure 3b), showing an almost monotonic decrease while increasing *T* from 800 to 900 °C (maximum In concentration of 14.64%); on the same graph, the data referred to the single-layer P10 (violet square) is demonstrating the reproducibility of the collected experimental data and at the same time excluding any major effects related to the multilayer approach (e.g., effect from underlying layers). A superlinear increase (Figure 3c) in the In incorporation is recorded while increasing the O-flux (fixed *T*_g_ = 900 °C and In-flux = 0.7 nm^–2^ s^–1^): in this case, despite the high *T*_g_, a maximum In incorporation of about 19.25% is obtained. In this case, the data collected on single layers deposited in a different PAMBE apparatus qualitatively confirm the dependence of In incorporation on the provided oxygen flux (Figure 3d). More in general, even if two different MBE experimental apparatus and various deposition parameters were employed, a comparison among all of the single layers reported in Table 1 (i.e., P02, P10, U09, U13, U19), allows to qualitatively confirm that the provided oxygen and the substrate temperature are the major growth parameters affecting the In incorporation in the (In_*x*_Ga_1–*x*_)_2_O_3_ alloy. These recorded dependencies of the synthesis parameters with respect to the In-flux incorporation are qualitatively similar to what has been previously observed during plasma-assisted MBE of κ-, β-, as well as α-(In_*x*_Ga_1–*x*_)_2_O_3_, alloys [*c*-plane sapphire substrates with and without β-Ga_2_O_3_(−201) nucleation layers for κ and β, *m*-plane sapphire substrate for α].^28,44−46^

### 3.2 Impact of In Content *x* on Structural Quality

#### 3.2.1 Surface Morphology

**Figure 4:**
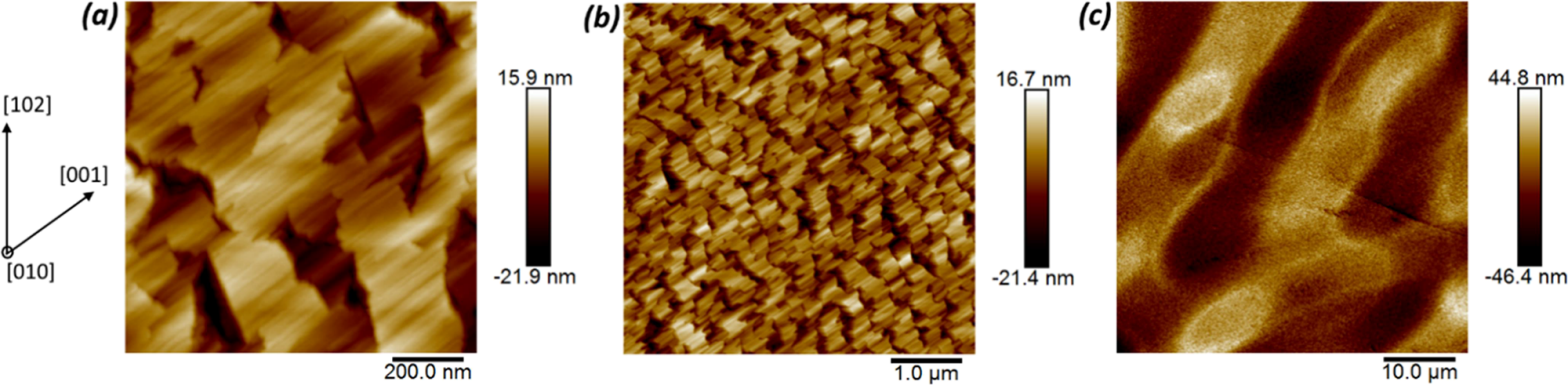
AFM topographs of the multilayer for 1 × 1, 5 × 5, and 50 × 50 μm^2^ scanned areas for (a), (b), and (c), respectively. Height scale and in-plane direction are indicated.

AFM on 1 × 1 and 5 × 5 μm^2^ area scans of the multilayer PM sample (Figure 4a,b) shows features already highlighted for other (010) homoepitaxial β-Ga_2_O_3_ layers, i.e., (110) and () facets visible as elongated features along [001] and deeper trenches almost orthogonal to them.^14,19^ The image with the largest scan area of 50 × 50 μm^2^ (Figure 4c) shows that some inhomogeneity is present in the multilayer. This is most likely related to the different growth conditions that were already shown to affect the quality of the deposited layers in the case of (010) β-Ga_2_O_3_ homoepitaxy.^14^

**Figure 5:**
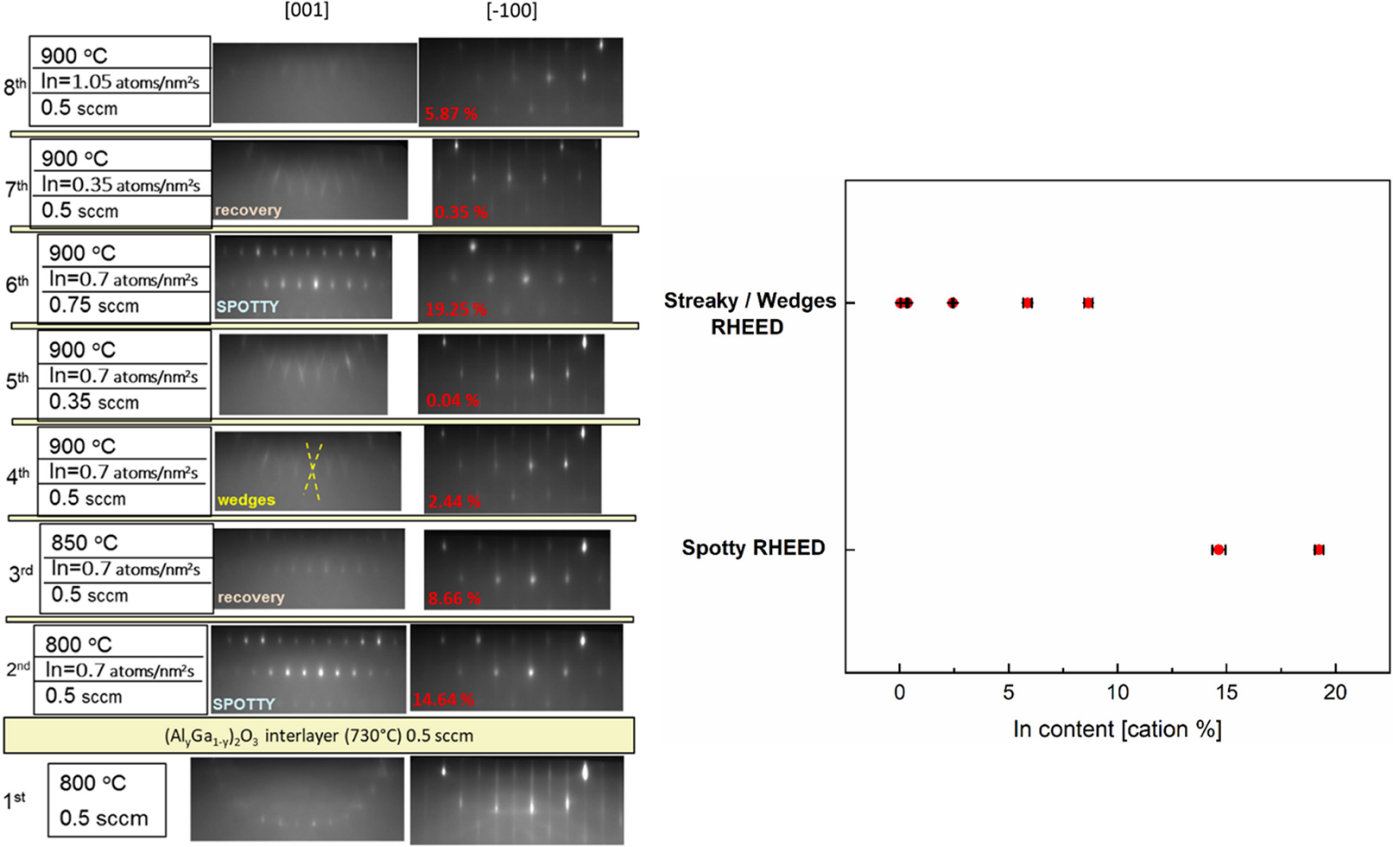
RHEED images from [001] and [−100] azimuths taken in situ after the deposition of every single β-(In_*x*_Ga_1–*x*_)_2_O_3_ layer of sample PM; a qualitative dependence of the RHEED pattern with respect to the In incorporation is also provided.

**Figure 6:**
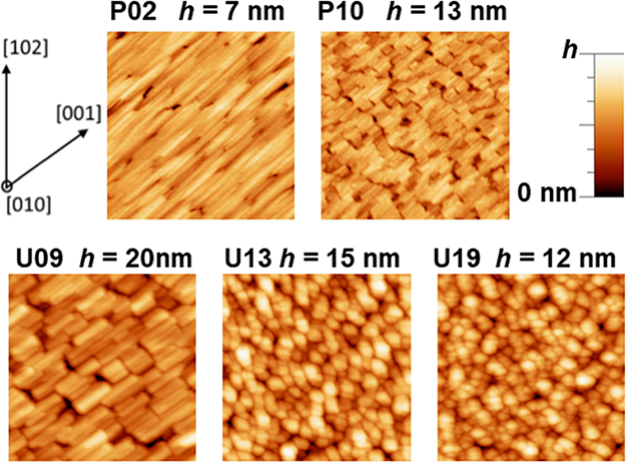
1 × 1 μm AFM images of the β-(In_*x*_Ga_1–*x*_)_2_O_3_ single layers (details in Table 1).

In Figure 5, the RHEED images taken *in situ* after the deposition of the individual layers of sample PM are reported. It is possible to identify a better quality of the RHEED images for an In content up to 8.66% (i.e., passage from wedges and streaks for the [001] and [−100] azimuths, respectively, to a spotty pattern). The presence of wedges in the RHEED images acquired along the [001] azimuth is related to the (110)-faceting^14^ of the (010)-oriented β-(In_*x*_Ga_1–*x*_)_2_O_3_ layers. The presence of these shallow facets is independently confirmed by the AFM images acquired for the single-layer samples (Figure 6). For *x* ≥ 0.13, the AFM single-layer morphology lacks oriented features and exhibits a grainy morphology. Nonetheless, we stress that it could be misleading to attribute the discussed morphology transition just to the content of In: both *x* and other growth parameters (e.g., *T*_g_, metal-to-oxygen flux ratio) can compete in determining the observed AFM morphologies.

#### 3.2.2 Phase Stability and Microstructure

**Figure 7:**
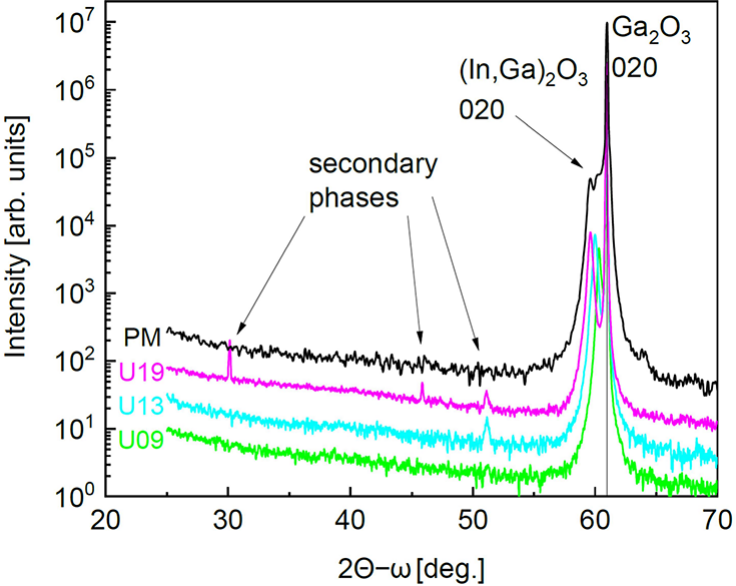
Exemplary wide-range XRD 2θ-ω scans are used to identify secondary phases.

All samples were investigated via XRD. The wide-range symmetric out-of-plane 2θ-ω scans of the multilayer sample PM and the single layers U09–U19, reported in Figure 7, show strong reflexes in the vicinity of the 020 β-Ga_2_O_3_ main substrate reflection, which are related to the β-(In_*x*_Ga_1–*x*_)_2_O_3_ layers and β-(Al_*y*_Ga_1–*y*_)_2_O_3_ marker layers. The absence of additional strong reflexes in the XRD of the PM multilayer suggests that all layers maintain a single monoclinic phase despite the wide range of In incorporation (as later confirmed by TEM). Therefore, differently from the growth on (100) β-Ga_2_O_3_ substrates,^22^ the MBE growth on the (010) orientation allows for In incorporation of at least as high as ≈ 19% for pure β-(In_*x*_Ga_1–*x*_)_2_O_3_ alloyed layers. Only for U13 and U19, weak additional reflexes can be observed, suggesting the possible formation of a minor fraction of secondary orientations or secondary phases for an In-content exceeding 10%. These secondary phases/orientations may cause the grainy morphology visible in the AFM images of these single layers (Figure 6). The appearance of possible secondary phases in the U single layers has not been further investigated; nevertheless, our results collected from two different MBE systems suggest that slightly different synthesis parameters could eventually result in small fractions of different phases from the pure monoclinic one for *x* > 0.1.

**Figure 8:**
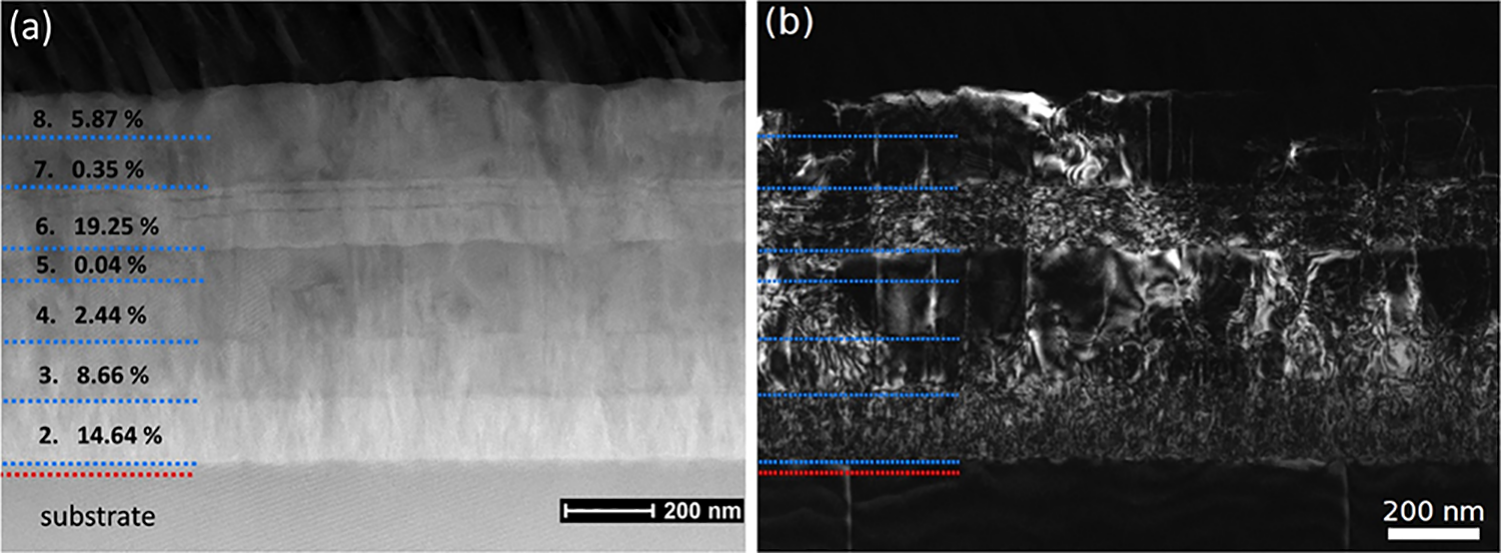
(a) HAADF STEM and (b) dark-field TEM overview images of the complete structure of sample PM, imaged in cross-section in the [001] projection of the Ga_2_O_3_ substrate.

**Figure 9:**
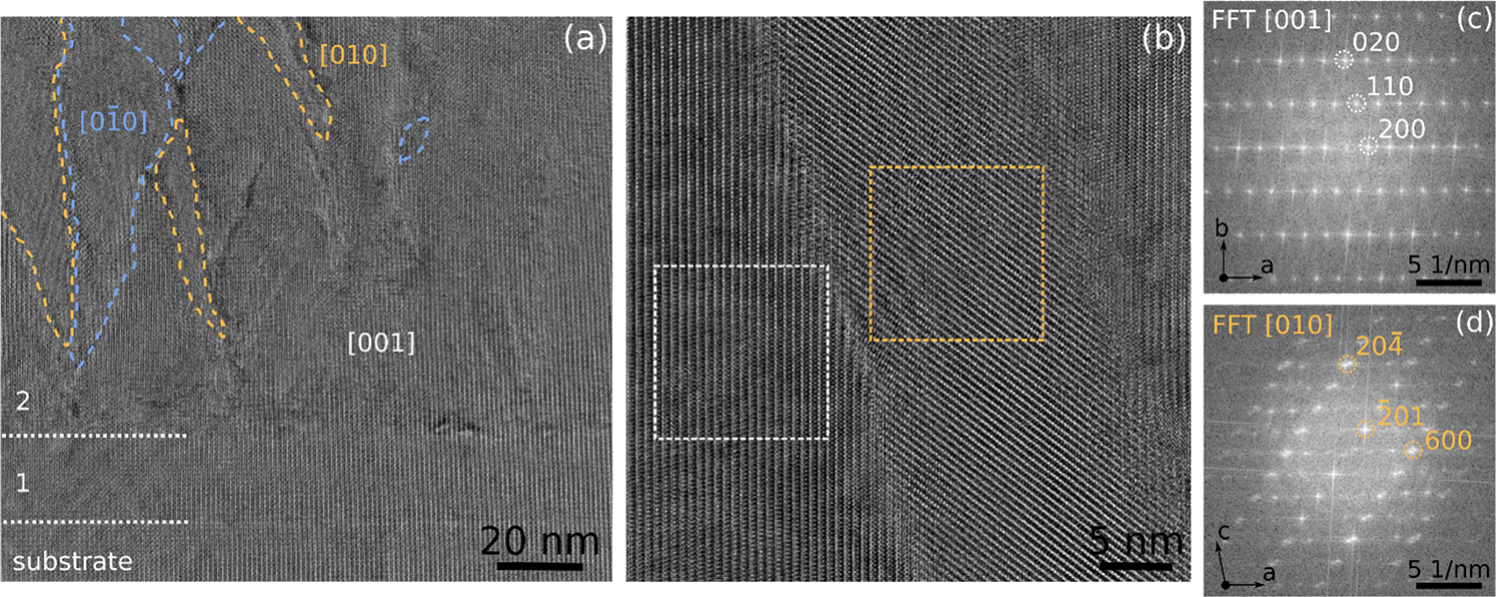
(a) HRTEM image of sample PM showing the formation of [010] and [] projected twinned grains in the 2nd layer with 14.64% In. (b) Higher magnification image of a [010]-projected grain (orange) embedded in the [001]-projected matrix (white), within (c) and (d) the FFTs of both areas.

The impact of In incorporation on the layer microstructure is analyzed in detail by means of TEM for each layer of the PM multilayer. For this purpose, the cross-sectional structure is viewed in the [001] projection. HAADF STEM and weak beam dark-field (WBDF) TEM overview images are presented in Figure 8a,b. In the HAADF image, the intensity is qualitatively proportional to the mean atomic number of the material. This allows us to distinguish the single layers with different compositions based on intensity, and the highest indium content layers estimated from SIMS (second and sixth) indeed appear the brightest. The contrast in each layer is not homogeneous though, and additional intensity effects can be caused by composition fluctuations, lattice defects, or differences in strain or orientation. The interface between subsequent layers starts out rather smooth but gets rougher as the number of deposited layers increases. The resulting surface also is rough with peak-to-valley distances on the order of a few nanometers but without the formation of clear facets with defined angles, as observed in the MBE growth of (010) β-Ga_2_O_3_.^14^ In the 19.25% layer, two horizontal dark intensity bands of ∼10 nm thickness can be observed. Higher magnification images of those regions showed no disruption of the monoclinic structure; therefore, it is not caused by phase separation. We rather suspect there was some unintentional change in growth parameter (e.g., fluctuation of plasma power) during deposition, which resulted temporarily in a lower indium content. The WBDF image on the right is produced by selecting only one diffraction spot and tilting the sample slightly off Bragg condition, which basically means defects will “light up” in the image. The brightest intensity in the highest indium-containing layers indicates the highest formation of defects there, i.e., lowering of the crystalline quality. The defects are often transferred into the overgrown layers, but an overall decrease in WBDF intensity with a lowering of the indium content shows a “recovery” in the crystal structure, consistent with the recovery observed in situ by RHEED. High-resolution TEM (HRTEM) in the high indium-containing parts of the PM multilayer structure (i.e., 14.64 and 19.25% In layers) was performed to investigate more deeply what is causing the extended defects formation. As illustrated in Figure 9a,b for the 14.4% In layer, grains with an HRTEM pattern differing from the expected [001] projected [(010)-oriented] structure are observed. From the fast Fourier transform (FFT) of such a grain, it is found that they correspond to a different projection of the monoclinic phase, namely, [010] projection with growth along (), as illustrated in Figure 9d. Domains of both twin orientations [010] and [], colored in light orange and blue, respectively, in Figure 9a, are found. They start to form in the second layer with 14.64% In and again in the sixth layer with 19.25% In and some penetrate through to the next layer or even further. However, on average they are disappearing as the structure gets overgrown with material of lower indium content. The twin domains appear slightly brighter in the HAADF STEM image, which seems to indicate that indium is segregating in those grains. This could explain the deviation of the out-of-plane lattice parameter to lower values for indium content >14% (Figure 11) since these are based only on (010)-oriented material.

Thus, despite maintaining the monoclinic structure for all investigated compositions, indium can be incorporated at least up to approximately 10% as a single-orientation/phase layer with low structural defectivity and without indium segregation.

### 3.3 Lattice Parameters and Anisotropic In-Plane Strain Relaxation

#### 3.3.1 Out-of-Plane Lattice Parameter b

**Figure 10:**
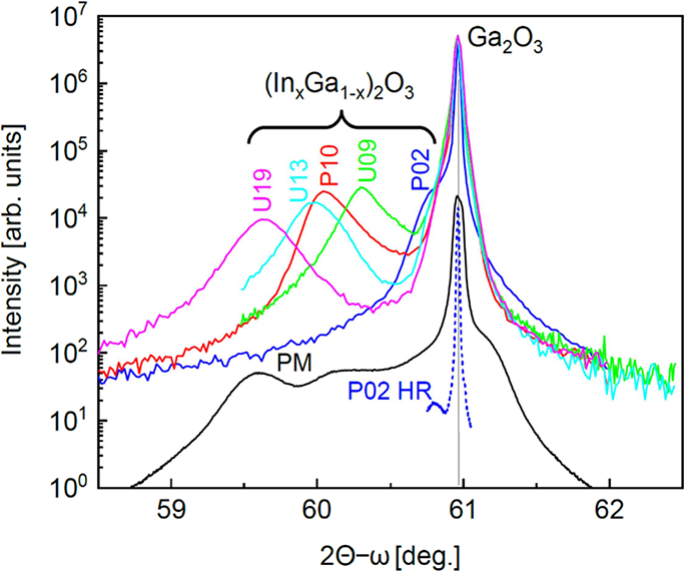
XRD 2θ-ω scans of the layer reflexes of all samples in the vicinity of the 020 β-Ga_2_O_3_ reflex.

Figure 10 shows detailed 2θ-ω scans in the vicinity of the 020 substrate reflex for all of the samples. For PM, the convolution of several reflexes at the left side of the substrate reflex is related to the β-(In_*x*_Ga_1–*x*_)_2_O_3_ layers with different compositions and lattice parameter *b* larger than that of pure β-Ga_2_O_3_. Only the position of the reflex at the lowest 2θ angle (layer with largest *b*, thus highest In content *x*) can be clearly determined. The single shoulder on the right-hand side of the substrate reflex should be assigned to the seven β-(Al_*y*_Ga_1–*y*_)_2_O_3_ marker layers (see Figures 1 and 2b), having a smaller *b*. With increasing In content, the corresponding XRD scans of the individually grown β-(In_*x*_Ga_1–*x*_)_2_O_3_ singe-layer samples exhibit, besides the substrate reflex, an additional broad reflex each at increasingly smaller 2θ angle, corresponding to increasingly larger *b*. To resolve the reflex position (shoulder) of the lowest In content sample, P02, we additionally show HRXRD.

**Figure 11:**
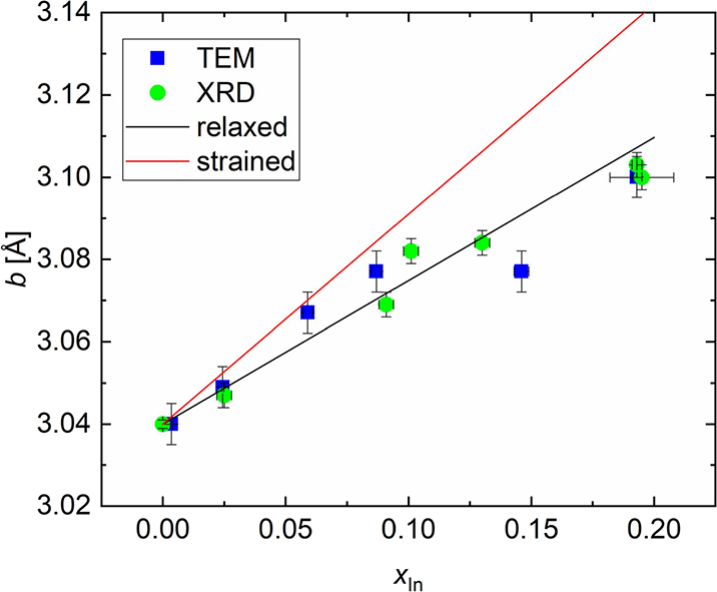
Lattice parameter *b* as a function of In content locally measured in sample PM by TEM diffraction patterns, as well as by XRD in the single layers. Black and red lines represent two extremes of fully relaxed and pseudomorphically strained β-(In_*x*_Ga_1–*x*_)_2_O_3_-layers using the relaxed lattice parameters published by Kranert et al.^26^ for β-(In_*x*_Ga_1–*x*_)_2_O_3_ powders and calculations of biaxially strained (010) layers from Oshima et al.,^47^ respectively. *b* = 3.040 Å was assumed consistently for β-Ga_2_O_3_ in line with our HRXRD measurements of the substrate 020 reflection.

**Figure 12:**
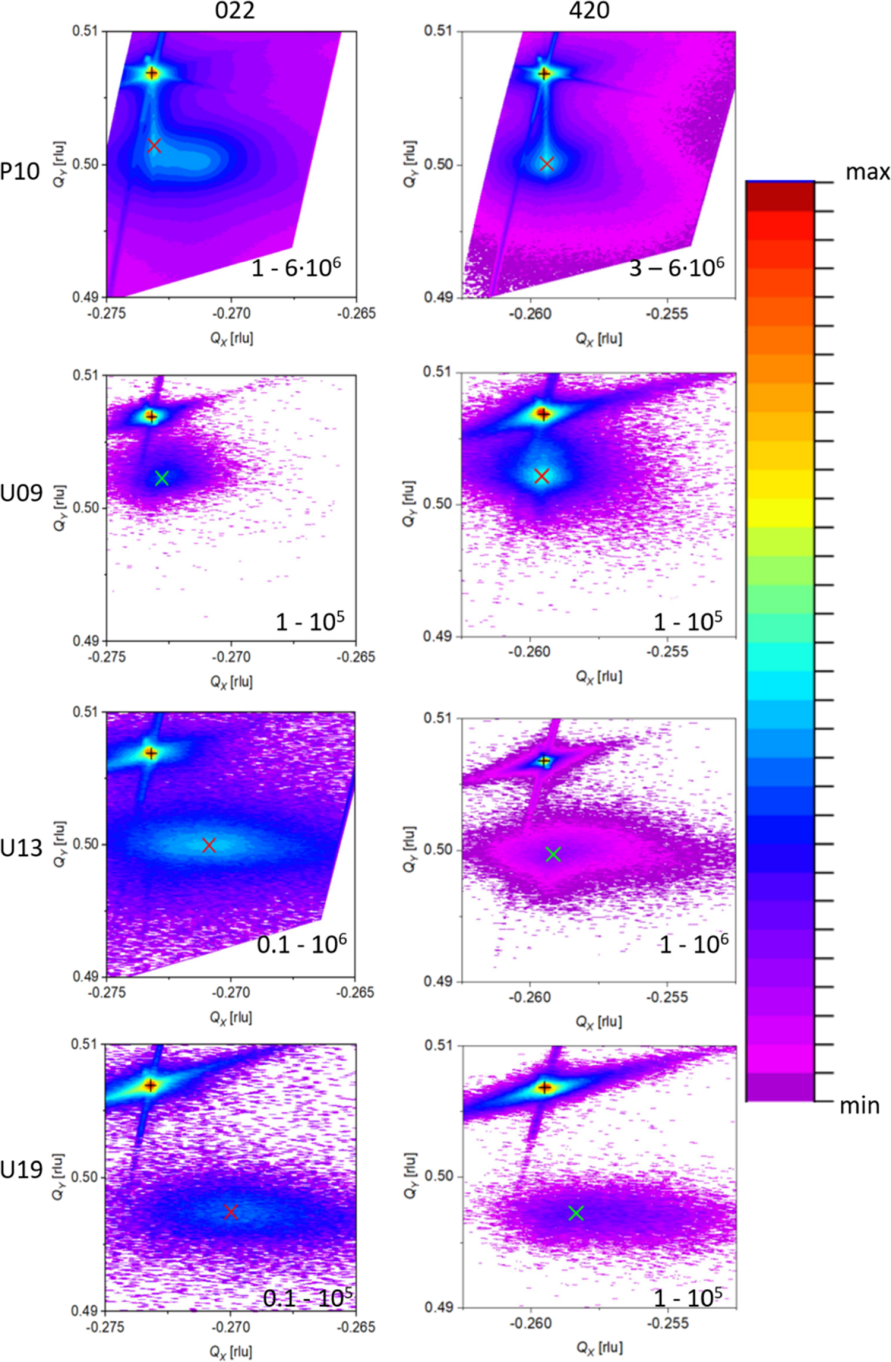
XRD reciprocal space maps of samples P10, U09, U13, and U19 taken for the 022 and 420 reflexes. The position of the sharp substrate reflex is marked by a “+,” whereas the broad reflex of the layer is marked by a “*x*”. The correspondence of the logarithmic color scale on the right side to minimum and maximum intensity is given in the maps.

Figure 11 shows the out-of-plane lattice parameter *b* of all β-(In_*x*_Ga_1–*x*_)_2_O_3_ layers (measured by HRXRD) as a function of In-content. For sample PM, *b* was locally measured in all individual β-(In_*x*_Ga_1–*x*_)_2_O_3_ layers by TEM-based selected area electron diffraction (SAED), placing a moveable aperture in the beamline such that only intensity from a specific β-(In_*x*_Ga_1–*x*_)_2_O_3_ is contributing to the diffraction image. For all single layers and the highest-In-containing layer of PM, *b* was extracted from the β-(In_*x*_Ga_1–*x*_)_2_O_3_ 020 reflex positions of XRD analysis (Figure 10). For reference, lines representing predicted values (based on literature as indicated) for fully relaxed or pseudomorphically strained films are also shown. The out-of-plane lattice parameters extracted by both methods show remarkable agreement and are approximately following the Vegard’s law. For layers with In content below 10% inside sample PM, *b* could be well represented by the pseudomorphically strained scenario, whereas the relaxed scenario seems to better apply for larger In content (further discussion on this aspect based on reciprocal space maps of single layers, see Figure 12).

#### 3.3.2 Coherent Growth and Anisotropic In-Plane Strain Relaxation

The larger lattice parameter of the β-(In_*x*_Ga_1–*x*_)_2_O_3_ layer compared to that of the β-Ga_2_O_3_ substrate is prone to result in a compressive strain of the layer. To shed light on the strain state of the layer, the single-layer samples were further investigated by XRD reciprocal space maps. Figure 12 shows reciprocal space maps for the 022 and 420 reflexes of the samples P10, U09–U19, corresponding to c* and a* in-plane directions, respectively, to resolve potential anisotropies.

**Figure 13:**
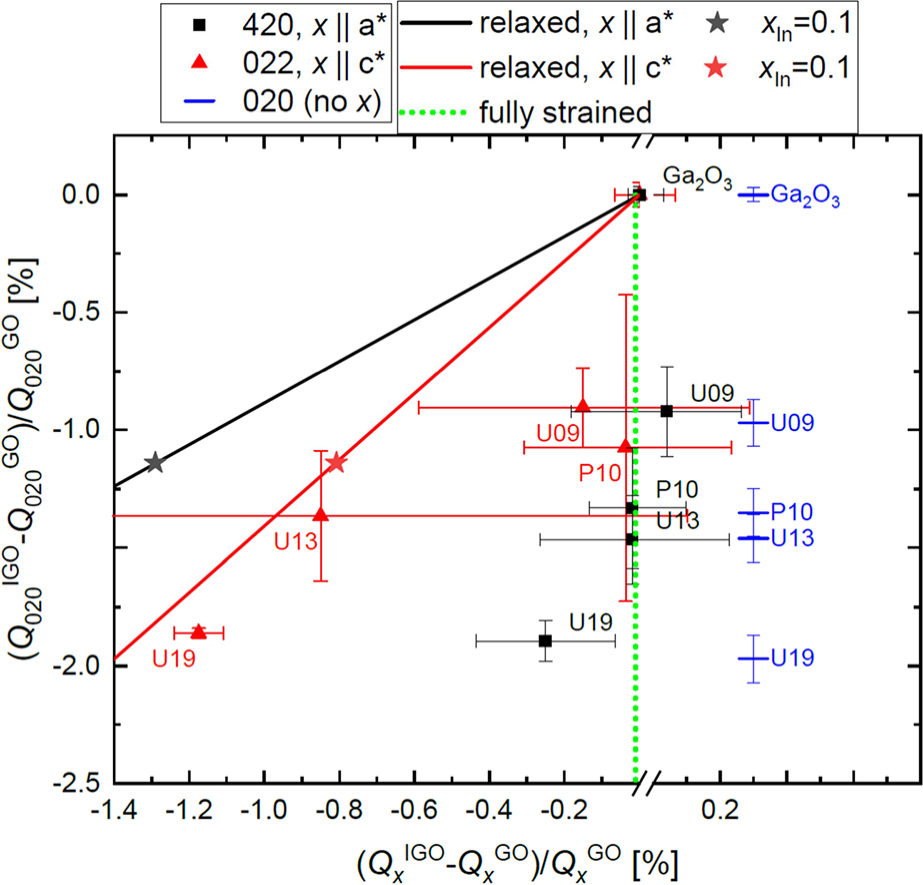
Summary of reciprocal space coordinates of 020, 420, and 022 β-(In_*x*_Ga_1–*x*_)_2_O_3_ reflexes in comparison to those of β-Ga_2_O_3_. The error bars reflect the full widths at half maximum values of the reflexes. Expected positions for the relaxed β-(In_*x*_Ga_1–*x*_)_2_O_3_ lattice (from Kranert et al.^26^) are shown as black and red lines for the 420 and 022 reflexes, respectively.

The positions of the substrate and layer reflexes are marked in Figure 12 and compiled in a systematic summary, shown in Figure 13, of strain relaxation for all single-layer samples. In this representation, the relative change of reciprocal space coordinates of the 020 (only vertical component), 022, and 420 reflexes with respect to those of the substrate is given for the out-of-plane (*b*) direction as well as in-plane directions (a* and c*), and the substrate reflex is at the origin (0, 0). Reference lines are drawn for pseudomorphically strained (vertical green dotted line) and fully relaxed scenarios (oblique solid lines). Due to different relative changes of *a* and *c* lattice parameters with increasing In content, two different lines are drawn, the black for the a* in-plane component and the red one for the c*. The star-shaped symbols indicate the expected values for relaxed β-(In_0.1_Ga_0.9_)_2_O_3_.^26^

**Figure 14:**
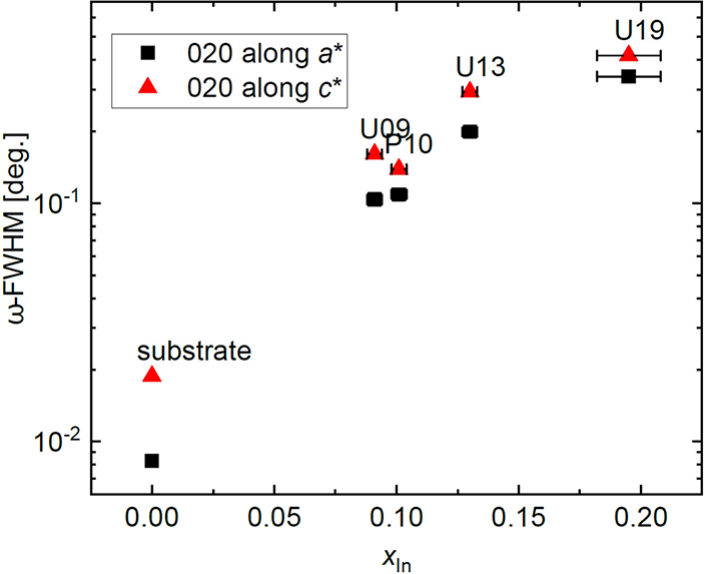
Full widths at half-maximum (FWHM) of the ω-rocking curve of the β-(In_*x*_Ga_1–*x*_)_2_O_3_ 020 reflex along c* and a* directions as indicated.

For each sample, the vertical components extracted from all three reflexes are in good (within ±0.1%) agreement and decrease with increasing In content, confirming a consistent set of data. The largest part of sample P10 with an In-content of 10% and 120 nm film thickness is coherently grown, indicated by coincindence of in-plane components in both directions (c* and a*) with those of the substrate (follow the green dotted line). This pseudomorpically strained scenario with compressive layer strain can also explain the increased out-of-plane lattice parameter *b* (due to the Poisson effect) that can be observed in the analysis of the 020 reflex (cf. Figures 10 and 11). Sample U09 with similar In content but a larger film thickness of 160 nm seems to exceed the critical thickness for strain relaxation as it shows some degree of relaxation and broader reflexes. For higher In content of about 13 and 19%, (almost complete) relaxation occurs only into the in-plane c* direction (022 reflexes agree approximately with the red reference line), whereas the layer remains almost pseudomorphically strained in the a* direction with partial relaxation for the 19% sample. The mosaic tilt, quantified by the full widths at half-maximum of the 020 ω-rocking curve, of the β-(In_*x*_Ga_1–*x*_)_2_O_3_ layers along both the c* and a* in-plane directions is shown in Figure 14 as a function of In content. It monotonically increases with increasing In content and shows consistently larger values along the c* than a* direction, consistent with the plastic relaxation occurring just for *x* > 0.1 at first along the c* direction.

The anisotropic in-plane strain relaxation found here, is similar to that observed for compressively strained α-(Al_*x*_Ga_1–*x*_)_2_O_3_ films on a-plane α-Al_2_O_3_ substrates exhibiting fast in-plane plastic relaxation into the [001] via dislocation glide on the r-plane slip system and almost pseudomorphic strain in the [−110] direction.^48,49^ The origin of the anisotropic strain relaxation in the (010)-oriented β-(In_*x*_Ga_1–*x*_)_2_O_3_ layers investigated in this work remains to be explored. We speculate, that an easier compression of the *a*-direction compared to that of the *c*-direction^50^ may facilitate the elastic strain in the a* direction. Practical consequences of the anisotropic strain relaxation are (i) conclusions on pseudomorphically strained layers should be drawn based on investigation of the relaxation in the c* rather than the a* direction, and (ii) misfit dislocations formed during plastic strain relaxation into the c* direction correspond to directional extended defects that will likely turn the fairly isotropic electrical conductivity,^51^ into one with anisotropic in-plane conductivity. For lateral field-effect transistors based on a β-(In_*x*_Ga_1–*x*_)_2_O_3_ channel, this would result in reduced channel mobility along the c* direction.

### 3.4 Prospect of Two-Dimensional Electron Gases in a (In_*x*_Ga_1–*x*_)_2_O_3_/(Al_*y*_Ga_1–*y*_)_2_O_3_ Heterostructures

A potential application of β-(In_*x*_Ga_1–*x*_)_2_O_3_ layers is the replacement of the β-Ga_2_O_3_ channel in a modulation-doped β-(Al_*y*_Ga_1–*y*_)_2_O_3_/β-Ga_2_O_3_ heterostructure to increase the confined sheet electron concentration *n*_S_. In such heterostructures, *n*_S_ is mainly limited by its approximately linear dependence on the conduction band offset Δ*E*_C_[channel, barrier] between the channel and barrier material.^52^ The Al content in the barrier material, which determines Δ*E*_C_[β-Ga_2_O_3_, β-(Al_*y*_Ga_1–*y*_)_2_O_3_], is limited by phase separation. For example, a 2DEG with *n*_s_ = 4.7 × 10^12^ cm^–2^ has been demonstrated for an Al content of *y* = 0.17 in the β-(Al_*y*_Ga_1–*y*_)_2_O_3_ barrier material, corresponding to an estimated Δ*E*_C_ ≈ 0.4 eV.^52^ Replacing β-Ga_2_O_3_ by β-(In_*x*_Ga_1–*x*_)_2_O_3_ in the channel can further increase Δ*E*_C_[channel, barrier] by Δ*E*_C_[β-(In_*x*_Ga_1–*x*_)_2_O_3_, β-Ga_2_O_3_] due to the lower lying conduction band of β-(In_*x*_Ga_1–*x*_)_2_O_3_. Theoretical predictions of Δ*E*_C_[β-(In_*x*_Ga_1–*x*_)_2_O_3_, β-Ga_2_O_3_] are ranging from 1.3 eV for *x* = 1,^33^ to 0.4 eV for an epitaxial, (100)-oriented interface with *x* = 0.12.^31^ Assuming the limitation of *x* = 0.10 for a low concentration of extended defects, coherently grown β-(In_*x*_Ga_1–*x*_)_2_O_3_ determined here, Δ*E*_C_[β-(In_*x*_Ga_1–*x*_)_2_O_3_, β-Ga_2_O_3_] would be limited to 0.13 or 0.33 eV. Thus, Δ*E*_C_[β-(In_0.1_Ga_0.9_)_2_O_3_, β-(Al_0.17_Ga_0.83_)_2_O_3_] would be limited to 0.53 or 0.73 eV, with the prospect to confine a *n*_S_ of up to 6.2 × 10^12^ or 8.6 × 10^12^ cm^–2^ instead of 4.7 × 10^12^ cm^–2^ for the case of a β-Ga_2_O_3_ channel. We believe these values to be a lower bound estimate since to confine the 2DEG, β-(In_0.1_Ga_0.9_)_2_O_3_ can be significantly thinner (few nm) than the ∼120 nm-thick layer (P10) investigated here, thus allowing for potentially higher In-content while maintaining coherent growth as well as providing a back-barrier to the underlying β-Ga_2_O_3_. Nevertheless, it should be considered that the increase of *n*_S_ may come at the expense of reduced channel mobility due to additional alloy scattering and reduced breakdown voltage because of the lower bandgap of β-(In_0.1_Ga_0.9_)_2_O_3_ compared to β-Ga_2_O_3_.

## 4 Summary and Conclusions

In this work, the growth and structural properties of monoclinic β-(In_*x*_Ga_1–*x*_)_2_O_3_ layers were explored to predict their prospects for the increase of sheet electron concentration *n*_S_ of 2DEGs confined in β-(In_*x*_Ga_1–*x*_)_2_O_3_/(Al_*y*_Ga_1–*y*_)_2_O_3_ heterostructures compared to state-of-the-art β-Ga_2_O_3_/β-(Al_*y*_Ga_1–*y*_)_2_O_3_ ones.

For this purpose, 60–180 nm-thick β-(In_*x*_Ga_1–*x*_)_2_O_3_ layers were grown by molecular beam epitaxy on β-Ga_2_O_3_ substrates in one of the most technologically relevant growth orientations of the monoclinic gallium oxide material system, i.e., (010). With qualitative agreement of the results from two different MBE systems, the collected data give practical experimental guidelines for the control of In content *x* by growth parameters and demonstrate the possibility of depositing high-quality β-(In_*x*_Ga_1–*x*_)_2_O_3_ layers free from secondary phases or orientations up to at least *x* = 0.1. With such an In content, the conduction band offset confining the 2DEG in β-(In_*x*_Ga_1–*x*_)_2_O_3_/(Al_*y*_Ga_1–*y*_)_2_O_3_ heterostructures could be at least 0.13 eV larger than in β-Ga_2_O_3_/(Al_*y*_Ga_1–*y*_)_2_O_3_ ones, with the prospect to potentially increase *n*_S_ from 4.7 × 10^12^ cm^–2^ for an Al content of *y* = 0.17 (ref (52)) to > 6.2 × 10^12^ cm^–2^.

The strain relaxation with respect to the underlying (010) β-Ga_2_O_3_ substrate was found to be markedly anisotropic, with faster relaxation into the c* direction than in the a* direction. The associated anisotropic dislocation formation could lead to anisotropic in-plane transport in electronic devices with β-(In_*x*_Ga_1–*x*_)_2_O_3_ channels as thick as our layers.

A 2DEG can be contained in significantly thinner β-(In_*x*_Ga_1–*x*_)_2_O_3_ layers than those studied here. Therefore, it is reasonable to assume that their reduced thickness allows for an increased In content, and thus stronger confinement of a higher *n*_S_, without reduction of structural quality.
